# Dual-Subpopulation Competitive Particle Swarm Optimization with Engineering Applications

**DOI:** 10.3390/biomimetics11020144

**Published:** 2026-02-13

**Authors:** Shuying Zhang, Yufei Zhang, Minghan Gao, Qiaohong Zhang, Yue Gao

**Affiliations:** 1 College of Computer Science and Technology, Beihua University, Jilin 132013, China; jlzhangsy@beihua.edu.cn; 2 College of Computer Science and Technology, Changchun University, Changchun 130022, China; zhangyf@ccu.edu.cn; 3 College of Information Science and Technology, Jinan University, Guangzhou 510632, China; 4 School of Computer Science and Engineering, Sun Yat-sen University, Guangzhou 510275, China

**Keywords:** swarm intelligence, particle swarm optimization, engineering optimization

## Abstract

Particle swarm optimization (PSO) is a widely used bio-inspired optimization algorithm, yet maintaining an effective balance between exploration and exploitation remains challenging. Most existing PSO variants rely on static or predefined regulation strategies, which restrict their adaptability to evolving search states and may lead to premature convergence or search stagnation. Inspired by division of labor and competitive selection mechanisms in biological populations, this paper proposes a dual-subpopulation competitive particle swarm optimization (DCPSO). In DCPSO, the population is explicitly partitioned into exploration and exploitation subpopulations with distinct search roles. A dynamic competition mechanism is designed to evaluate recent search progress, based on which stagnated particles are adaptively migrated between subpopulations, enabling flexible reallocation of computational resources during the optimization process. Experimental results on the CEC2017 benchmark suite demonstrate that DCPSO consistently outperforms standard PSO and several representative state-of-the-art algorithms, achieving statistically significant improvements on the majority of benchmark functions, particularly on hybrid and composition problems. Additional experiments on engineering design problems further verify the robustness, convergence stability, and practical effectiveness of DCPSO.

## 1. Introduction

Swarm intelligence (SI) optimization algorithms constitute a class of computational methods inspired by collective behaviors observed in natural systems, such as bird flocking [1], fish schooling [2], and insect foraging [3]. In these systems, complex global intelligence emerges from local interactions among simple individuals without centralized control. This decentralized and self-organizing nature makes SI algorithms particularly suitable for solving complex and nonlinear optimization problems [4], and has led to their widespread application in areas such as engineering design [5,6], control systems [7,8], and machine learning [9,10].

Particle swarm optimization (PSO) is one of the most well-known and widely used SI algorithms [11]. It models social information sharing within a population, where particles adjust their trajectories according to both personal experience and social learning. Owing to its fast convergence speed and ease of implementation, PSO has achieved considerable success in a variety of optimization tasks [12,13,14]. Nevertheless, similar to many bio-inspired algorithms, PSO faces a fundamental challenge: achieving an effective balance between exploration and exploitation [15].

Exploration refers to probing new regions of the search space to avoid being trapped in local optima, whereas exploitation focuses on intensively refining promising regions discovered during exploration to obtain high-quality solutions [16]. Due to the dynamic nature of the optimization process, the relative importance of these two conflicting objectives varies over time and is often jointly influenced by problem characteristics and the current search state. Excessive exploration may result in slow convergence, while excessive exploitation frequently leads to premature convergence to local optima. Therefore, maintaining an appropriate balance between exploration and exploitation is crucial to the overall performance of particle swarm optimization.

Numerous PSO variants have been proposed to mitigate the exploration–exploitation trade-off, including parameter control strategies [17,18,19], topology modification methods [20,21,22], and multi-strategy hybridization techniques [23,24,25]. These approaches primarily regulate particle behaviors by adjusting coefficients, modifying information-sharing patterns, or integrating additional search operators. Although such designs can influence the intensity of exploration and exploitation, they operate mainly at the strategy or interaction level. The overall population organization typically remains unchanged throughout the search, which may limit the algorithm’s ability to adapt when the global search demand shifts significantly.

To further enhance behavioral diversity, multi-population or role-based PSO variants  [26,27,28] explicitly divide the swarm into subgroups with different search responsibilities. Compared with single-strategy regulation, these methods introduce functional differentiation within the population. However, in most existing designs, the partitioning scheme and role assignments are fixed once initialized, and the relative influence of each subpopulation is not dynamically adjusted according to their actual search contributions. As a result, even though heterogeneous behaviors coexist, the structural configuration of the swarm lacks an internal mechanism for self-reorganization when one search mode becomes temporarily dominant or ineffective.

To overcome these limitations, we propose a dual-subpopulation competitive particle swarm optimization (DCPSO) framework that introduces structural adaptation at the population level. Inspired by division of labor and competitive selection in natural systems, DCPSO differentiates exploration and exploitation roles while enabling their relative dominance to evolve during the search process. Division of labor is implemented through role-specific update strategies, whereas competitive selection is realized via a performance-driven migration mechanism. By periodically evaluating recent search contributions using a sliding-window metric, stagnated particles are adaptively transferred between subpopulations. Through this competition-driven reorganization process, the swarm structure continuously self-adjusts in response to changing optimization demands, achieving a dynamic and state-aware coordination between exploration and exploitation.

The main contributions of this paper are summarized as follows:

- A dynamically adaptive dual-subpopulation framework is proposed, in which exploration and exploitation roles are explicitly differentiated while allowing population composition to evolve during the search process. Unlike static PSO variants, the proposed framework enables structural self-adjustment based on search state.
- A dynamic competition and migration mechanism is introduced, where a sliding-window-based winning-strength metric is used to evaluate subpopulation performance and adaptively reallocate particles. This mechanism enables resource redistribution in response to the search state.
- Dedicated update strategies are developed for each subpopulation, employing elite-restricted neighborhood learning for exploitation and fitness-ranking-based dimensional learning for exploration. These tailored strategies reinforce the complementary functions of the two subpopulations and improve the overall search efficiency.

The remainder of this paper is organized as follows: Section 2 introduces the standard PSO algorithm and its advanced variants. Section 3 describes the proposed DCPSO framework in detail. Section 4 presents numerical experimental results and comparative analyses. Section 5 reports simulation results on three engineering optimization problems. Finally, Section 6 concludes the paper and discusses directions for future research.

## 2. Preliminaries and Related Work

### 2.1. Particle Swarm Optimization

PSO is a population-based stochastic optimization algorithm originally proposed by Kennedy and Eberhart. It is inspired by the collective movement and information-sharing mechanisms observed in social organisms such as bird flocks and fish schools. In PSO, a population of particles collaboratively explores a continuous search space in order to locate optimal or near-optimal solutions.

Let *N* denote the population size and *D* the dimensionality of the search space. Each particle i∈{1,2,…,N} is associated with a position vector $x_{i}^{t}\in R^{D}$ and a velocity vector $v_{i}^{t}\in R^{D}$ at iteration *t*. The position represents a candidate solution, while the velocity determines the direction and magnitude of the particle’s movement in the search space.

During the optimization process, each particle retains a memory of its best historical position, referred to as the personal best and denoted by $pbest_{i}$. In addition, information about the best position found by the entire population, known as the global best and denoted by gbest, is shared among all particles. Based on this information, the velocity and position of particle *i* are updated according to

(1) $$v_{i}^{t+1}=\omega v_{i}^{t}+c_{1}r_{1}\left(pbest_{i}-x_{i}^{t}\right)+c_{2}r_{2}\left(gbest-x_{i}^{t}\right),$$

(2) $$x_{i}^{t+1}=x_{i}^{t}+v_{i}^{t+1},$$

where ω is the inertia weight controlling the influence of the previous velocity, $c_{1}$ and $c_{2}$ are acceleration coefficients corresponding to the cognitive and social components, respectively, and $r_{1}$ and $r_{2}$ are vectors of independent random variables uniformly distributed in ${\left[0,1\right]}^{D}$.

The canonical PSO is attractive due to its simple structure and low computational cost. However, it is well known to suffer from premature convergence and rapid loss of population diversity, particularly in multimodal or high-dimensional optimization problems, where particles may quickly cluster around suboptimal regions. Although adjusting control parameters such as inertia weight and acceleration coefficients can partially influence the exploration–exploitation balance, parameter tuning alone cannot fundamentally reflect the collective search state of the population or reorganize its structural behavior once diversity has significantly decreased. These limitations have motivated the development of more adaptive and structurally enhanced PSO variants.

### 2.2. Related Work

PSO has inspired extensive research aimed at improving its search behavior across diverse optimization problems. Existing studies are commonly grouped into three categories: parameter control–based methods, topology-oriented designs, and hybrid multi- strategy approaches.

#### 2.2.1. Parameter Control–Based PSO

Parameter control methods regulate particle dynamics by adjusting key coefficients, such as inertia weight, acceleration parameters, or velocity bounds, in order to influence the search trajectory during evolution. For example, Yang et al. [29] proposed a nonlinear time-varying inertia weight scheme combined with low-discrepancy initialization to enhance global coverage. Song et al. [30] introduced a fractional-order adaptive velocity parameter to perturb particle updates based on evolutionary states. Minh et al. [31] incorporated an additional velocity component governed by a decreasing function to gradually shift from exploration to exploitation. Meng et al. [32] dynamically adjusted paradigm proportions and contraction coefficients, while Nayeem et al. [33] integrated adaptive inertia regulation with guidance from the Grey Wolf Optimizer.

These approaches refine particle movement through adaptive parameter scheduling and have demonstrated effectiveness across various problem settings. Since their regulation primarily operates on update coefficients, the overall population organization and role distribution typically remain unchanged during the search process. As optimization progresses, the collective search behavior is therefore mainly influenced through parameter variation rather than structural reconfiguration.

#### 2.2.2. Topology-Oriented PSO

Topology-oriented PSO variants modify interaction structures among particles to regulate information exchange and collective learning. Li et al. [21] proposed pyramid PSO (PPSO), in which particles are hierarchically organized into multiple fitness-based layers. Information exchange occurs both within layers and across adjacent layers, enabling multi-level learning and diversity preservation. Jin et al. [28] further extended PPSO by incorporating an adaptive constraint-handling mechanism that propagates feasibility information along the hierarchical structure. Flori et al. [22] developed QUAPSO, which partitions the swarm into exploration and exploitation sub-populations with differentiated update rules and multi-position representations. Hu et al. [34] introduced an adaptive elite–common partitioning strategy with cooperative coevolutionary interactions, while Zhou et al. [35] designed a region-based solution selection mechanism that defines neighborhoods around discovered optima to encourage distributed exploration.

These approaches primarily regulate how information is propagated within the swarm through structured communication patterns. In most cases, group assignments are determined by predefined or fitness-based rules, and adjustments mainly occur at the interaction level. In contrast, DCPSO adaptively reallocates particles between functional roles over time through performance-driven migration, enabling dynamic adjustment of population composition rather than solely modifying communication structure.

#### 2.2.3. Hybrid and Multi-Strategy PSO

Hybrid PSO approaches integrate multiple optimization mechanisms within a unified framework to exploit complementary search behaviors. Li et al. [36] proposed a multi-component PSO algorithm that maintains a strategy pool of distinct PSO variants coordinated by a leader-learning mechanism. Adamu et al. [37] hybridized PSO with an enhanced crow search algorithm by introducing time-varying transfer functions and opposition-based learning. Şenel et al. [38] combined PSO with the Grey Wolf Optimizer by probabilistically refining a subset of particles, while Shi et al. [39] embedded genetic crossover and mutation operators into the PSO updating process. Lin et al. [40] incorporated simulated annealing to probabilistically accept inferior solutions and enhance local escape capability.

These frameworks enrich search behavior by introducing external optimization components. DCPSO differs in that adaptability is achieved through internal competition between role-differentiated subpopulations rather than through integration of additional metaheuristics. The additional computational cost in DCPSO mainly arises from periodic performance evaluation and migration operations, remaining moderate compared with hybrid strategies that coordinate multiple optimization modules.

## 3. Dual-Subpopulation Competitive Particle Swarm Optimization

### 3.1. Dual-Subpopulation Competitive Framework

The core idea of DCPSO is to maintain two subpopulations with complementary search roles and to dynamically adjust their relative influence according to recent search progress. Intuitively, when one search mode demonstrates stronger improvement, more computational resources are gradually allocated to that mode through particle migration, allowing the swarm structure to evolve along with the optimization process.

At initialization, the population is evenly divided into an exploitation subpopulation (*E*) and an exploration subpopulation (*X*), each consisting of N/2 particles, where *N* denotes the total population size. Although equal partitioning is adopted for simplicity, the framework itself does not rely on strict balance. Due to the adaptive migration mechanism, moderate unequal initial splits were observed to have negligible influence on the final performance, as the population composition is continuously adjusted during evolution.

**Figure 1:**
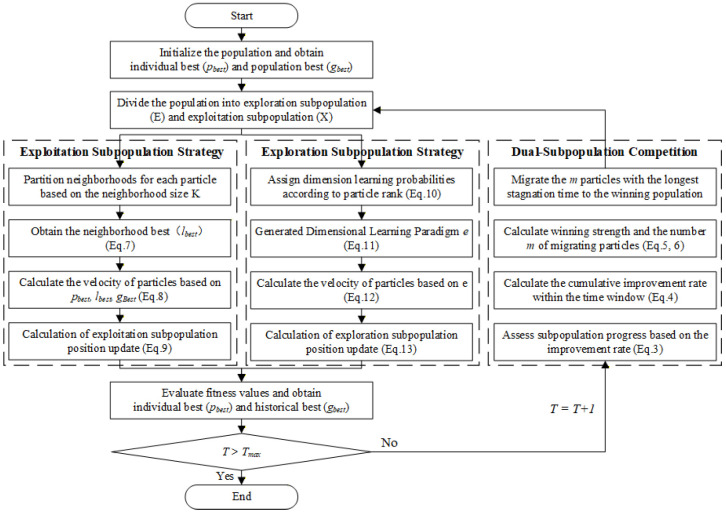
Overall flowchart of DCPSO.

The overall flowchart of DCPSO is illustrated in Figure 1. As illustrated in Figure 1, the competition–migration cycle is embedded within each iteration of the algorithm, forming a closed-loop structural adaptation process. At each iteration, the two groups evolve independently according to their respective update strategies, thereby reinforcing different search behaviors. After the evolution step, a population-level competition mechanism is triggered.

Specifically, the recent search efficiency of each group is evaluated through subpopulation progress assessment (Section 3.1.1), based on which the dominant and inferior subpopulations at the current stage are identified. Subsequently, a winning intensity indicator (Section 3.1.2) is computed to quantify the dominance degree of the superior group, which further determines the number of particles to be migrated. Finally, through a stagnated-particle migration mechanism (Section 3.1.3), the dominant subpopulation takes over a portion of the most severely stagnated particles from the inferior one. Through this competitive interaction, the population structure is dynamically adjusted to accommodate the evolving requirements of the search process.

#### 3.1.1. Subpopulation Progress Evaluation

This subsection quantifies the recent evolutionary effectiveness of each subpopulation. An improvement-rate metric based on a sliding window is employed. At iteration *t*, the improvement rate of subpopulation k∈{E,X} is defined as

(3) $$\rho_{k}\left(t\right)=\frac{\left|\left\{i\in P_{k}|f\left(x_{i}^{t}\right)<f\left(x_{i}^{t-1}\right)\right\}\right|}{|P_{k}|},$$

where $P_{k}$ denotes the particle set of subpopulation *k*, and $f(x_{i}^{t})$ represents the fitness value of particle *i* at iteration *t*.

To mitigate the influence of stochastic fluctuations in a single iteration, a sliding window of length *W* is adopted. The cumulative improvement rate within this window is computed as

(4) $$S_{k}\left(t\right)=\sum_{\tau =t-W+1}^{t}\rho_{k}\left(\tau \right),\;k\in \left\{E,X\right\}.$$

The window length *W* controls the trade-off between responsiveness and stability. A moderate value was selected based on empirical evaluation, and its influence is further analyzed in the parameter sensitivity study presented in Section 4.3. The group exhibiting a higher cumulative improvement rate is regarded as the dominant one at the current stage.

#### 3.1.2. Winning Intensity Indicator

To characterize the dominance degree of the winning group over the losing one, a winning intensity indicator is introduced. Based on the cumulative improvement capability of each group, the winning intensity at iteration *t* is defined as

(5) $$\eta \left(t\right)=\frac{|S_{E}\left(t\right)-S_{X}\left(t\right)|}{S_{E}\left(t\right)+S_{X}\left(t\right)+\varepsilon },$$

where ε is a small positive constant introduced to avoid division by zero. The value η(t)∈[0,1) reflects the relative performance gap between the two groups: larger values indicate stronger dominance, whereas values close to zero imply nearly balanced competition.

#### 3.1.3. Stagnated Particle Migration Mechanism

The proposed competition achieves adaptivity by adjusting the population structure through particle migration. The competition and migration procedure is performed at every iteration, ensuring timely structural adjustment in response to ongoing search dynamics.

When a subpopulation is identified as dominant within the sliding window, particles in the inferior group are ranked according to their stagnation degree, defined as the number of consecutive iterations without fitness improvement. The top *m* most severely stagnated particles are selected and reassigned to the dominant group. The migration size is adaptively determined as

(6) $$m=\left\lceil \eta \left(t\right)\cdot m_{\max}\right\rceil ,$$

where $m_{\max}$ is a predefined upper bound on the number of migratable particles, typically set as a small fraction of the subpopulation size to avoid excessive structural perturbations.

When the two groups exhibit comparable performance (i.e., η(t) falls below a predefined threshold), a small number of particles randomly exchange their strategy labels between subpopulations. This lightweight perturbation helps prevent structural stagnation and maintains population diversity.

It should be emphasized that migration only changes the strategy affiliation of particles; their positions, velocities, and historical best information remain unchanged, thereby preserving search continuity and memory.

### 3.2. Exploitation Subpopulation Strategy

This subsection describes the exploitation strategy, which aims to conduct stable and refined local search. Neighborhood learning and a constriction factor are jointly employed. To prevent excessive amplification of global-best information that may lead to rapid population homogenization, neighborhood interactions are restricted within the exploitation subpopulation, and the influence of the global best is intentionally weakened.

#### 3.2.1. Neighborhood-Based Learning

An extended ring topology is adopted to construct the local cooperation mechanism. For each exploitation particle *i*, its neighborhood $N_{i}$ consists of *K* adjacent particles on each side in the ring. Only neighbors belonging to the exploitation group are retained, forming the effective neighborhood ${\tilde{N}}_{i}$.

The neighborhood best position $lbest_{i}$ is defined as

(7) $$lbest_{i}=\arg\min_{j\in \left\{i\right\}\cup {\tilde{N}}_{i}}f\left(pbest_{j}\right).$$

By confining information flow to locally high-quality substructures, this elite-restricted learning mechanism enhances exploitation efficiency while suppressing premature convergence caused by global-best dominance.

#### 3.2.2. Velocity and Position Update

Let $x_{i}^{t}\in R^{D}$ and $v_{i}^{t}$ denote the position and velocity of particle *i* at iteration *t*, respectively. The personal best position is denoted by $pbest_{i}$, and the global best position by gbest. A constriction-factor-based velocity update is adopted to enhance stability:

(8) $$v_{i}^{t+1}=\chi \left(v_{i}^{t}+c_{1}r_{1}\left(pbest_{i}-x_{i}^{t}\right)+c_{2}r_{2}\left(lbest_{i}-x_{i}^{t}\right)+c_{3}r_{3}\left(gbest-x_{i}^{t}\right)\right),$$

where χ is the constriction factor, and $c_{1}$, $c_{2}$, and $c_{3}$ are acceleration coefficients corresponding to individual, neighborhood, and global guidance, respectively. Although local neighborhood learning dominates the exploitation process, a weakened global-best component is retained to provide global directional consistency and prevent excessive isolation of local neighborhoods. This mild global guidance helps maintain convergence coherence without inducing rapid population homogenization.

The position is updated as

(9) $$x_{i}^{t+1}=x_{i}^{t}+v_{i}^{t+1}.$$

### 3.3. Exploration Subpopulation Strategy

The exploration strategy aims to maintain global diversity and continuously supply promising candidate solutions to the exploitation process. Unlike the exploitation strategy, it does not explicitly rely on global or neighborhood best positions. Instead, a dimension-wise random learning mechanism is employed to promote information recombination across particles and dimensions, thereby alleviating dimension coupling in high- dimensional spaces.

#### 3.3.1. Dimension Learning Probability

To induce differentiated exploration behaviors, a fitness-ranking-based dimension learning probability is introduced. Let $N_{X}$ denote the size of the exploration subpopulation, and let $r_{i}\in \left\{1,2,\ldots ,N_{X}\right\}$ represent the fitness rank of particle *i*, where a smaller rank indicates better fitness. The dimension learning probability is defined as

(10) $$P_{i}=P_{\min}+\left(P_{\max}-P_{\min}\right)\frac{\exp\;\left(\alpha \frac{r_{i}-1}{N_{X}-1}\right)-1}{\exp(\alpha )-1}.$$

Here, $P_{\min}$ and $P_{\max}$ are the lower and upper bounds of the learning probability, respectively, and α is the learning factor. This monotonic mapping assigns lower learning probabilities to better-performing particles, preserving their search structures, while encouraging poorer-performing ones to explore unknown regions through more aggressive recombination.

#### 3.3.2. Dimension Learning and Update Mechanism

Based on $P_{i}$, a learning vector $e_{i}$ is constructed. For each dimension *d*, particle *i* selects two particles from the exploration subpopulation with probability $P_{i}$ and inherits the *d*-th component of the personal best position from the better one; otherwise, it retains its own component:

(11) $$e_{i,d}=\left\{\begin{array}{cc} p_{\kappa ,d}, & \mathrm{if}\;\mathrm{rand}<P_{i},\;\kappa =\arg\min_{j\in \{a,b\}}f\left(p_{j}\right),\\ p_{i,d}, & \mathrm{otherwise}. \end{array}\right.$$

The velocity update is given by

(12) $$v_{i}^{t+1}=\omega \left(t\right)v_{i}^{t}+cr\left(e_{i}-x_{i}^{t}\right),$$

where ω(t) is a time-decreasing inertia weight. The position update follows

(13) $$x_{i}^{t+1}=x_{i}^{t}+v_{i}^{t+1}.$$

Through dimension-wise exemplar recombination, the exploration subpopulation continuously generates structurally diverse candidate solutions, thereby effectively complementing the exploitation process. The dimension-wise recombination is performed independently for each coordinate, resulting in an additional computational cost proportional to O(D) per particle, which is comparable to standard velocity update operations. Therefore, the mechanism remains scalable for high-dimensional problems.

Conceptually, the dimension-wise exemplar selection shares similarity with differential evolution–style coordinate recombination. However, instead of constructing trial vectors through differential mutation, the proposed mechanism directly leverages personal best information within the exploration subpopulation, maintaining compatibility with the PSO updating framework.

### 3.4. Computational Complexity

This subsection analyzes the computational complexity of the proposed DCPSO. Let *N* denote the population size, *D* the problem dimensionality, *T* the maximum number of iterations, *K* the neighborhood radius in the exploitation subpopulation, *W* the sliding window length, and $C_{f}$ the cost of a single fitness evaluation.

At each iteration, both subpopulations perform particle updates. For the exploitation subpopulation, neighborhood-best identification incurs a cost of O(NK), while velocity and position updates require O(ND). For the exploration subpopulation, dimension-wise learning and state updates also require O(ND). The competition mechanism introduces additional overheads: computing improvement rates is O(N), and fitness-based ranking in the exploration subpopulation, together with stagnated-particle selection for migration, incurs O(NlogN). The sliding-window accumulation can be implemented with a running sum, resulting in O(1) amortized cost per iteration.

In addition, fitness evaluation dominates the algorithmic cost, requiring $O(NC_{f})$ per iteration. Therefore, the overall time complexity per iteration is

(14) $$O\left(NC_{f}+ND+NK+N\log N\right),$$

and the total computational complexity over *T* iterations is

(15) $$O\left(T\left(NC_{f}+ND+NK+N\log N\right)\right).$$

When the fitness evaluation is computationally expensive, the overall cost is dominated by the $O(TNC_{f})$ term; otherwise, the additional overhead introduced by neighborhood learning and competitive adaptation remains moderate and scales linearly or near-linearly with the population size.

For comparison, the per-iteration time complexity of standard PSO is $O(NC_{f}+ND)$, dominated by velocity–position updates and fitness evaluations. AFMPSO introduces additional forgetting and centroid-guided operations that scale linearly with population size and dimension, thus remaining $O(N\log NC_{f}+ND)$ with a slightly larger constant factor. SDPSO requires extra strategy payoff evaluation and probability updating, incurring an additional O(N) overhead while maintaining the same asymptotic order, i.e., $O(NC_{f}+ND)$.

In contrast, DCPSO further incorporates neighborhood learning and competition-based migration, leading to $O(NC_{f}+ND+NK+N\log N)$ per iteration. Since *K* is a small constant and fitness evaluation typically dominates the computational cost in practical applications, the overall complexity remains effectively governed by $O(NC_{f})$, indicating that the additional structural adaptation overhead is moderate and asymptotically comparable to standard and multi-strategy PSO variants.

### 3.5. Algorithmic Procedure

Based on the proposed dual-subpopulation competitive framework and the corresponding exploitation and exploration strategies, the overall procedure of DCPSO is summarized in Algorithm 1.
**Algorithm 1:** Dual-Subpopulation Competitive Particle Swarm Optimization
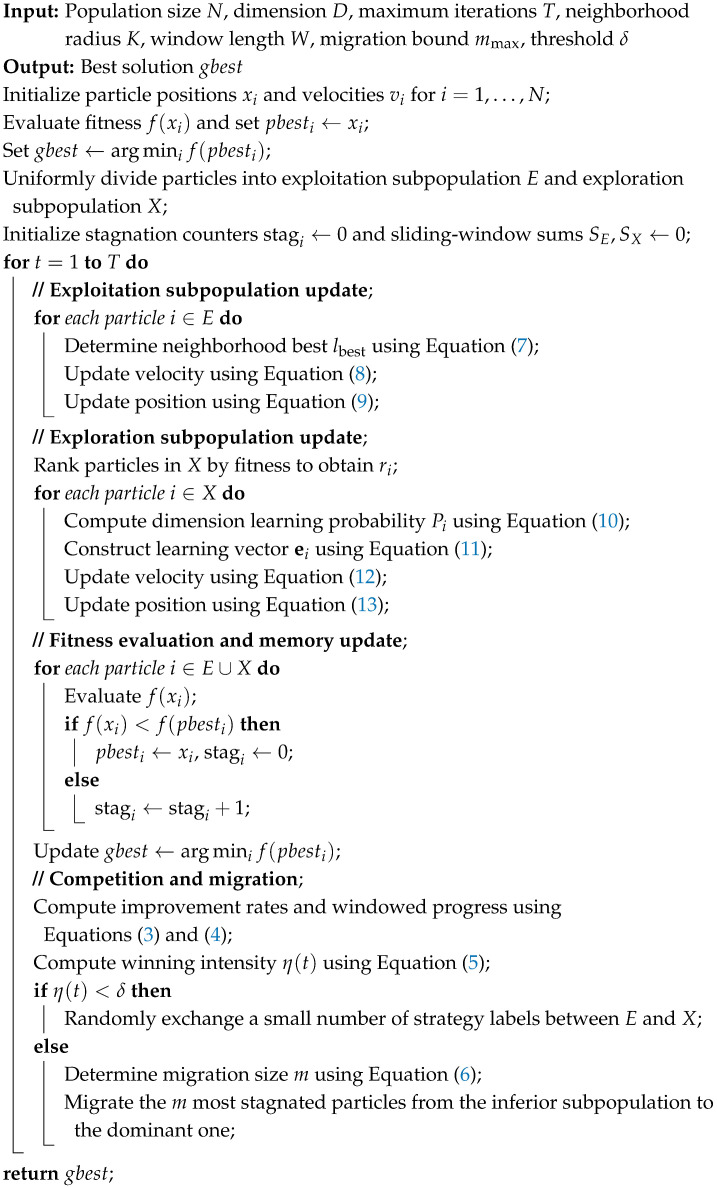


## 4. Experiments on Benchmark Functions

In this section, extensive experiments on benchmark functions are conducted to systematically investigate the performance of the proposed DCPSO algorithm. The experimental study is divided into three independent stages. First, a comprehensive performance comparison is carried out using the widely adopted CEC2017 benchmark suite [41] against several state-of-the-art PSO variants, including numerical results, convergence behaviors, and statistical significance tests. Second, a sensitivity analysis is performed to examine the influence of key algorithm parameters. Finally, an ablation study is conducted to isolate and verify the contributions of the specific algorithmic components introduced in the framework.

### 4.1. Benchmark Settings

#### 4.1.1. Test Environment

All simulation experiments are carried out in the MATLAB 2024a environment. The platform runs on a workstation equipped with the Windows 11 (25H2) operating system. The hardware configuration includes an Intel(R) Core(TM) i7-14700 (2.10 GHz) processor and 32 GB of system memory.

#### 4.1.2. Benchmark Problems

The CEC2017 benchmark test suite is selected as the test platform due to its wide acceptance and diverse problem characteristics. A notable feature of this benchmark is the extensive use of rotation and shift operations, which significantly increase variable coupling and non-separability. As a result, algorithms cannot easily locate the global optimum by optimizing individual dimensions independently.

The CEC2017 test suite consists of 29 benchmark functions, which can be categorized into four groups according to different search characteristics. Unimodal functions F1 and F3 contain only one global optimum without local optima. These functions are mainly used to evaluate the convergence speed and exploitation capability of algorithms. Multimodal functions F4 to F10 include multiple local optima. They are designed to assess the global exploration ability of algorithms and their capability to escape from local optima and avoid premature convergence. Hybrid functions F11 to F20 divide the variables into several groups, where each group is governed by a different basic function. This setting simulates complex real-world problems in which variables exhibit heterogeneous characteristics and requires algorithms to balance exploration and exploitation. Composition functions F21 to F30 are constructed by combining multiple basic functions using weighted schemes. They exhibit highly complex landscapes with numerous local traps and strong asymmetry, providing a rigorous test for an algorithm’s ability to balance global search and local refinement in complex search spaces.

#### 4.1.3. Parameter Settings

The parameters involved in DCPSO and other state of the art comparison algorithms are divided into common parameters and algorithm specific parameters.

For the common parameters, widely used settings in the literature are adopted. The population size is fixed at N=100, and the maximum number of iterations is set to T=1000, resulting in a maximum of 100,000 fitness evaluations. The problem dimension of the CEC2017 benchmark functions is set to D=30. Following common practice in evolutionary computation, each algorithm is independently executed 30 times on every benchmark function. This number of independent runs is widely adopted in the literature to ensure statistical reliability and to mitigate the influence of stochastic variability. The mean error and standard deviation are recorded for performance evaluation to reduce the influence of randomness.

For the algorithm-specific parameters, all comparison algorithms employ the parameter values recommended by their original authors to ensure fairness. The detailed parameter configurations of DCPSO and the baseline algorithms are listed in Table 1. The control parameters of DCPSO are fixed according to the empirical settings obtained from the parameter sensitivity analysis presented in Section 4.3. No additional tuning was performed for individual benchmark functions, ensuring fair and consistent comparisons across all experiments.

### 4.2. Performance Comparison on CEC2017

To comprehensively evaluate the effectiveness of DCPSO, comparative experiments are conducted on all 29 benchmark functions. Five state of the art PSO variants are selected as competitors. The detailed numerical results, including mean performance, standard deviation, and ranking statistics, are summarized in Section 4.2.1. The convergence behaviors on selected functions are illustrated in Section 4.2.2. In addition, the statistical significance analysis is employed in Section 4.2.3 to examine the statistical significance of the performance differences.

#### 4.2.1. Numerical Results

Table 2 reports the numerical performance of all compared algorithms on the 29 benchmark functions from the CEC2017 test suite in terms of mean error, standard deviation, and overall ranking. The results indicate that DCPSO demonstrates a consistent performance advantage over the baseline methods. Specifically, DCPSO achieves the best fitness values on 20 of the 29 test functions and attains the lowest average rank of 1.448, which is superior to all competing algorithms.

Among the baseline methods, ACEPSO ranks second with an average ranking of 3.034, followed by SDPSO with 3.517, MPSO with 3.724, AFMPSO with 4.690, VPPSO with 5.034, and the canonical PSO, which performs the worst with an average rank of 6.552. In addition to its dominant ranking performance, DCPSO also yields lower mean errors and smaller standard deviations on the majority of benchmark functions, indicating not only higher solution accuracy but also improved robustness and stability.

The performance advantage of DCPSO is more evident on the hybrid function set (F11–F20) and the composition function set (F21–F30), which are characterized by heterogeneous variable groupings and highly complex landscape structures. These functions typically require alternating phases of global exploration and refined local search, as different regions of the search space may demand different search intensities during evolution. The dual-subpopulation competitive framework allows exploration and exploitation tendencies to operate in parallel, while their relative influence is adaptively adjusted according to recent improvement trends. Such structural flexibility may help maintain diversity in early stages and reinforce local refinement when promising regions emerge. This complementary interaction mechanism could provide additional adaptability when addressing heterogeneous and dynamically shifting landscapes, thereby contributing to the observed performance trends in these complex problem categories.

#### 4.2.2. Convergence Behavior

**Figure 2:**
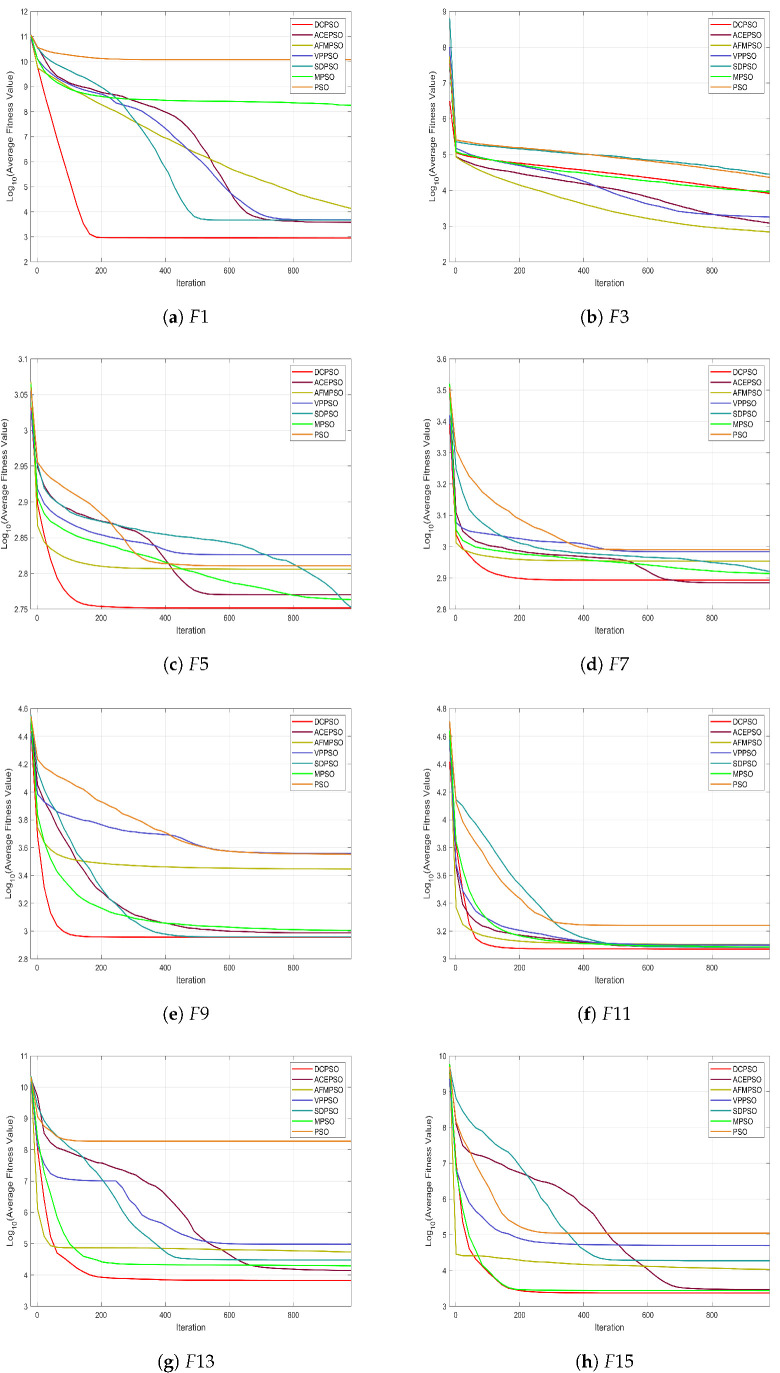

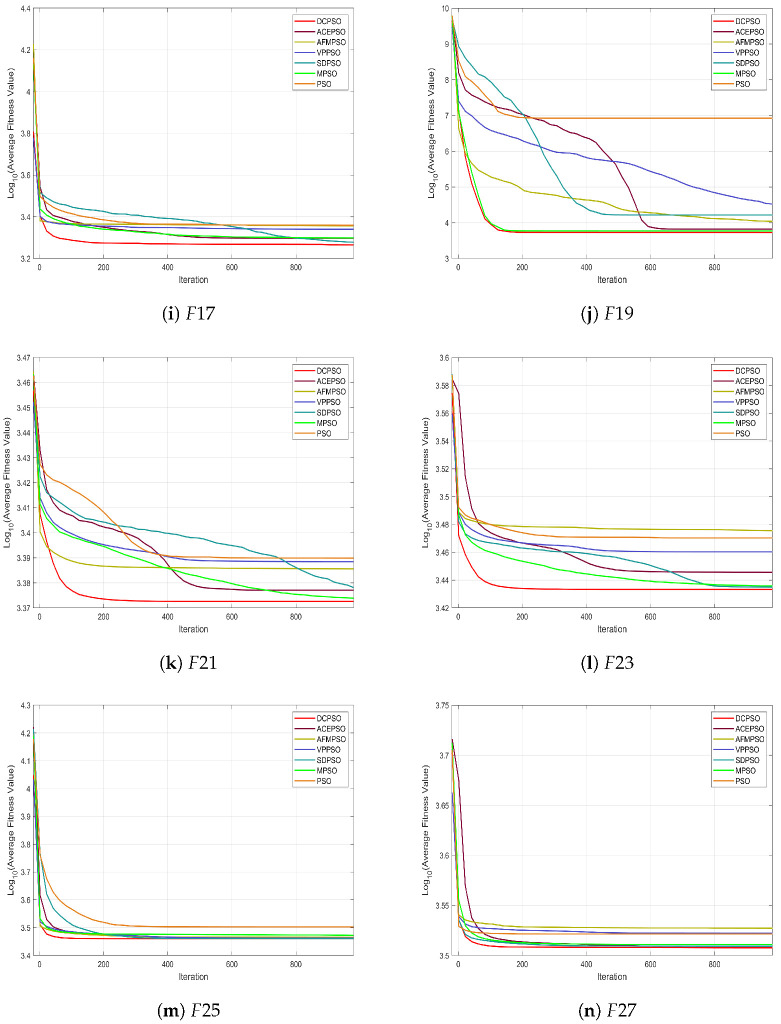
Average convergence trends of DCPSO and 6 comparison algorithms on selected CEC2017 functions.

To further evaluate the search dynamics of DCPSO, the average convergence curves over 30 independent runs are recorded on the odd-numbered benchmark functions of the CEC2017 test suite. The corresponding convergence curves are shown in Figure 2.

DCPSO exhibits favorable convergence behavior in terms of both convergence speed and final solution quality. On 12 out of the 15 selected benchmark functions, DCPSO converges to better fitness values within the given computational budget. In most cases, DCPSO achieves a faster reduction in fitness during the early and middle stages of the optimization process and maintains superior performance throughout the search.

It is worth noting that on function F3 (shown in Figure 2b), DCPSO exhibits relatively slower convergence compared with some competitors. Function F3 in CEC2017 is a unimodal problem with strong variable coupling introduced by rotation operations. Since DCPSO maintains a persistent exploration subpopulation and applies restrained global-best influence within the exploitation group, its search process may remain slightly more conservative in fully unimodal landscapes where rapid centralized convergence is beneficial. This behavior reflects a trade-off inherent in maintaining structural diversity, which is more advantageous for complex multimodal and composite functions but may result in less aggressive convergence on simpler unimodal cases.

In contrast, several baseline algorithms converge more slowly or suffer from premature stagnation, particularly on benchmark functions with complex search landscapes. These behaviors indicate limitations in balancing exploration and exploitation during the optimization process.

In addition, the convergence curves of DCPSO are notably smooth, suggesting stable search dynamics without abrupt oscillations. This smooth convergence behavior indicates that DCPSO maintains a controlled and consistent search process, thereby achieving an effective balance between exploration and exploitation.

#### 4.2.3. Statistical Significance Analysis

To further assess the statistical significance of the performance differences between DCPSO and the baseline algorithms, the Wilcoxon signed-rank test is employed [44]. As a nonparametric paired statistical test, the Wilcoxon signed-rank test does not rely on any distributional assumptions and is well suited for performance comparison of stochastic optimization algorithms on the same benchmark problems.

In this study, the Wilcoxon signed-rank test is conducted independently for each benchmark function (function-wise comparison) based on the paired fitness values obtained from 30 independent runs, with the significance level set to 0.05. For each benchmark function, pairwise comparisons are performed between DCPSO and each competing algorithm. The statistical test results are summarized in Table 3, where the symbols “+”, “≈”, and “−” indicate that DCPSO performs significantly better than, statistically equivalent to, or significantly worse than the corresponding algorithm, respectively.

As shown in Table 3, DCPSO achieves a dominant number of statistically significant wins over all baseline algorithms. Specifically, DCPSO records 25 wins and 4 losses against ACEPSO, 26 wins, 1 tie, and 2 losses against AFMPSO, 27 wins and 2 losses against VPPSO, 25 wins, 2 ties, and 2 losses against SDPSO, and 25 wins with 3 ties and only 1 loss against MPSO. In comparison with the canonical PSO, DCPSO achieves statistically significant improvements on all 29 benchmark functions, resulting in 29 wins and no losses.

The consistently high win ratios across all pairwise comparisons indicate that the observed performance advantages of DCPSO are statistically robust rather than caused by random variations. These statistical findings are in strong agreement with the numerical results and convergence analyses presented earlier. The overall comparison results further confirm that DCPSO consistently outperforms the baseline algorithms on the CEC2017 benchmark suite, demonstrating its robustness and effectiveness across a wide range of optimization problems.

### 4.3. Parameter Sensitivity Analysis

In DCPSO, two key parameters play an important role in controlling the search behavior, namely the sliding window length *W* used for subpopulation progress evaluation and the neighborhood size *K* adopted in the exploitation subpopulation. To examine the influence of these parameters on the algorithm performance, a parameter sensitivity analysis is conducted. When one parameter is analyzed, all other parameters are kept unchanged to ensure the fairness and reliability of the experimental results.

The sliding window length *W* determines the extent to which historical iterations are considered in evaluating subpopulation performance. Five different values are investigated, including 1, 5, 10, 15, and 20. The experimental results presented in Table 4 indicate that the choice of *W* has a noticeable impact on the performance of DCPSO. When *W* is set to a small value, the competition mechanism becomes overly sensitive to short-term fluctuations, which may lead to instability in the population structure. Conversely, excessively large values of *W* reduce the responsiveness to performance changes, thereby weakening the adaptability of the algorithm. According to the average ranking results, DCPSO achieves the lowest average rank when W=10, suggesting that this value provides a favorable balance between responsiveness and stability. Therefore, W=10 is selected as the recommended setting for DCPSO.

The neighborhood size *K* controls the rate of information sharing within the exploitation subpopulation. Five different values ranging from 1 to 5 are examined. As reported in Table 5, the results show that the algorithm performance is more sensitive when *K* takes smaller values. This can be attributed to the limited information exchange among particles when *K* is too small, which may slow down the convergence process. In contrast, a larger neighborhood size facilitates information sharing but may reduce population diversity and increase the risk of premature convergence, leading to a gradual degradation in performance. Based on the average ranking results, DCPSO achieves its best performance when K=3. Consequently, K=3 is adopted as the default neighborhood size in DCPSO.

Overall, although the two key parameters have a noticeable influence on the performance of DCPSO, they do not alter the algorithm’s significant performance advantage over the competing methods reported in Section 4.2. The main sensitivity findings can be summarized as follows:

- A moderate sliding window length (W=10) provides a good balance between responsiveness and stability. Very small values may lead to overly frequent structural fluctuations, while excessively large values may weaken adaptivity.
- An intermediate neighborhood size (K=3) facilitates sufficient information sharing within the exploitation subpopulation without excessively reducing diversity.
- Within the tested ranges, DCPSO demonstrates stable relative performance ranking, indicating a certain degree of robustness to moderate parameter variation.

From a practical perspective, when applying DCPSO to new optimization problems, it is recommended to start with the default settings (W=10, K=3) and adjust them moderately if the problem exhibits extremely noisy dynamics (where slightly larger *W* may improve stability) or requires faster local convergence (where slightly larger *K* may enhance information sharing). In most standard benchmark and engineering scenarios, extensive parameter tuning is not necessary.

### 4.4. Ablation Study

To analyze the contribution of different components in DCPSO, ablation experiments are conducted on selected CEC2017 benchmark functions. Three degraded variants are constructed by selectively removing key components, including DCPSO-X without the exploration subpopulation strategy, DCPSO-E without the exploitation subpopulation strategy, and DCPSO-DC without the dual-subpopulation competitive mechanism. All other algorithmic settings are kept unchanged to ensure a fair comparison. The experimental results are summarized in Table 6.

Overall, DCPSO achieved the best performance across all test functions. Removing the exploration strategy leads to noticeable performance degradation on most benchmarks, while removing the exploitation strategy results in a more severe decline, with substantial performance losses observed on many functions. When the competitive mechanism is disabled, DCPSO-DC also exhibits inferior performance compared with the full DCPSO, but generally outperforms DCPSO-X and DCPSO-E, indicating an intermediate level of effectiveness. In addition, all ablation variants consistently and significantly outperform the canonical PSO. These results indicate that each component contributes positively to the overall performance, and that the integration of exploration, exploitation, and adaptive competition is essential for achieving the superior performance of DCPSO.

## 5. Simulation on Engineering Optimization Problems

To assess the effectiveness and robustness of the proposed DCPSO algorithm, three representative constrained engineering optimization problems are considered in this section, including the three-bar truss design problem, the gear train design problem, and the pressure vessel design problem. These problems are commonly adopted in the literature due to their nonlinear objective functions, complex constraint conditions, and discrete or mixed-variable characteristics, which pose substantial challenges for population-based optimization algorithms.

### 5.1. Problem Formulations

#### 5.1.1. Three-Bar Truss Design

**Figure 3:**
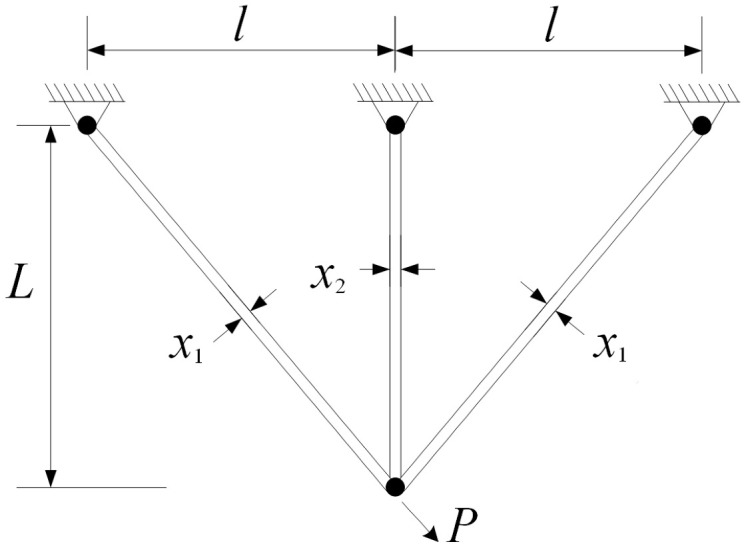
Schematic of the three-bar truss structure.

The three-bar truss design problem is a classical structural optimization problem that has been extensively studied in the context of constrained optimization. The objective is to minimize the total structural weight while satisfying stress and geometric constraints. This problem is characterized by a narrow feasible region and strong nonlinear constraints, which significantly increase the difficulty of locating feasible and high-quality solutions. The structure of the three-bar truss is illustrated in Figure 3.

The design variable vector is defined as

(16) $$x=[x_{1},x_{2}],$$

where $x_{1}$ and $x_{2}$ denote the cross-sectional areas of the truss members.

The objective function is formulated as

(17) $$\min f\left(x\right)=\left(2\sqrt{2}x_{1}+x_{2}\right)\times 100.$$

The nonlinear constraints are expressed as

(18) $$\begin{array}{cc} g_{1}\left(x\right) & =\frac{2(\sqrt{2}x_{1}+x_{2})}{\sqrt{2}x_{1}^{2}+2x_{1}x_{2}}-2\leq 0,\\ g_{2}\left(x\right) & =\frac{2x_{2}}{\sqrt{2}x_{1}^{2}+2x_{1}x_{2}}-2\leq 0,\\ g_{3}\left(x\right) & =\frac{2}{\sqrt{2}x_{1}+x_{2}}-2\leq 0, \end{array}$$

with variable bounds

(19) $$0\leq x_{1},\;x_{2}\leq 200.$$

#### 5.1.2. Gear Train Design

**Figure 4:**
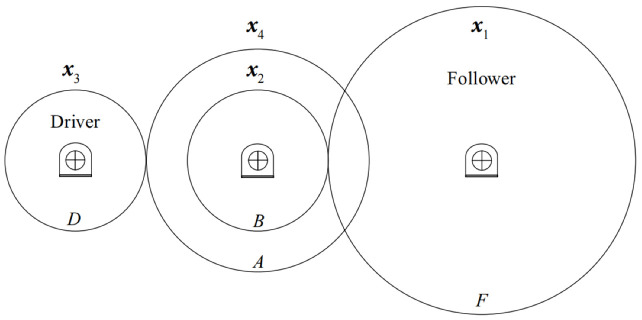
Schematic of the gear train design problem.

The gear train design problem is a representative discrete optimization problem frequently employed to evaluate the performance of algorithms on integer-valued decision variables. The objective is to determine the number of teeth of four gears such that the resulting gear ratio closely matches a predefined target value. Due to the discrete nature of the design variables and the nonlinear form of the objective function, the search space is highly discontinuous, which poses challenges for conventional continuous optimization techniques. The structure of the three-bar truss is illustrated in Figure 4.

The design variables are defined as

(20) $$x=[x_{1},x_{2},x_{3},x_{4}],$$

where each variable represents the number of gear teeth and takes integer values.

The objective function is defined as

(21) $$\min f\left(x\right)={\left(\frac{1}{6.931}-\frac{x_{3}x_{2}}{x_{1}x_{4}}\right)}^{2}.$$

The design variables are constrained by

(22) $$12\leq x_{i}\leq 60,\;x_{i}\in Z,\;i=1,2,3,4.$$

Solutions violating the constraints are penalized to restrict the search within the feasible region.

#### 5.1.3. Pressure Vessel Design

**Figure 5:**
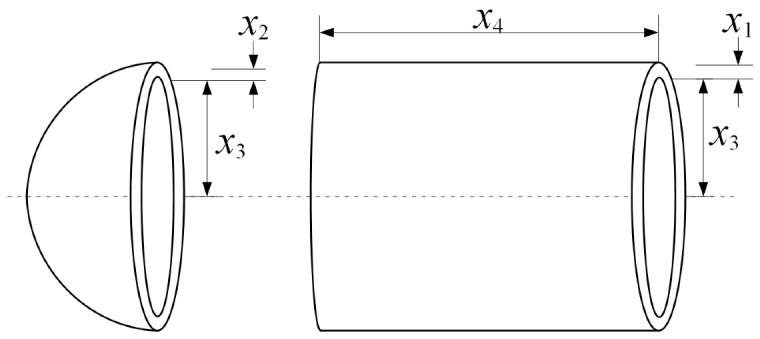
Schematic of the pressure vessel design problem.

The pressure vessel design problem is a well-known nonlinear constrained optimization problem originating from mechanical engineering design. The objective is to minimize the total fabrication cost while satisfying strength, volume, and dimensional constraints. This problem involves mixed discrete and continuous variables, making it particularly challenging for optimization algorithms to efficiently balance exploration and exploitation. The schematic representation of the vessel structure is shown in Figure 5.

The design variable vector is defined as

(23) $$x=[x_{1},x_{2},x_{3},x_{4}],$$

where $x_{1}$ and $x_{2}$ are discrete thickness variables with increments of 0.0625, $x_{3}$ represents the inner radius, and $x_{4}$ denotes the length of the cylindrical section.

The objective is to minimize the total fabrication cost, which is formulated as

(24) $$\begin{array}{cc} \min f(x)=\; & 0.6224x_{1}x_{3}x_{4}+1.7781x_{2}x_{3}^{2}\\ \; & +3.1661x_{1}^{2}x_{4}+19.84x_{1}^{2}x_{3}. \end{array}$$

The constraints are given by

(25) $$\begin{array}{cc} g_{1}\left(x\right) & =-x_{1}+0.0193x_{3}\leq 0,\\ g_{2}\left(x\right) & =-x_{2}+0.00954x_{3}\leq 0,\\ g_{3}\left(x\right) & =-\pi x_{3}^{2}x_{4}-\frac{4}{3}\pi x_{3}^{3}+1296000\leq 0,\\ g_{4}\left(x\right) & =x_{4}-240\leq 0, \end{array}$$

with variable bounds

(26) $$\begin{array}{c} 0\leq x_{1},x_{2}\leq 100,\\ 10\leq x_{3},x_{4}\leq 200. \end{array}$$

### 5.2. Experimental Settings

To ensure a fair and objective comparison, the proposed DCPSO algorithm and the competing algorithms are evaluated under identical experimental conditions. The comparison algorithms include ACEPSO, AFMPSO, VPPSO, SDPSO, MPSO, and the standard PSO. For all algorithms, the population size and the maximum number of iterations are set to 100 and $1.0\times 10^{5}$, respectively. To reduce the influence of stochastic effects, each algorithm is independently executed 30 times. The best mean and standard deviation (Std.) of the obtained solutions, along with the corresponding optimization variables of the best solutions, are recorded to assess optimization performance and robustness.

### 5.3. Results and Discussion

Table 7, Table 8 and Table 9 present the experimental results for the three-bar truss design problem, the gear train design problem, and the pressure vessel design problem, respectively. As shown in these tables, the proposed method achieves accurate optimal solutions and demonstrates superior overall performance compared with several state-of-the-art PSO variants, particularly in terms of the mean and standard deviation of the obtained results.

In the gear train design problem, the relatively low standard deviation observed across multiple independent runs indicates that DCPSO consistently attains lower objective function values, reflecting its enhanced robustness in discrete optimization scenarios. For the pressure vessel design problem, which involves mixed discrete–continuous variables and multiple nonlinearar constraints, DCPSO is able to reliably identify feasible solutions with reduced fabrication costs. Moreover, the algorithm exhibits stable convergence behavior and effectively avoids premature convergence, highlighting its suitability for addressing complex engineering optimization tasks.

Overall, the experimental results on these constrained engineering optimization problems confirm the robustness and effectiveness of the proposed algorithm. Its consistent performance across problems with diverse characteristics further suggests its strong potential for practical engineering applications.

## 6. Conclusions and Future Work

This paper proposed a dual-subpopulation competitive particle swarm optimization framework, termed DCPSO, to enhance the adaptive balance between exploration and exploitation in particle swarm optimization. In DCPSO, the population is explicitly partitioned into exploration and exploitation subpopulations with distinct functional roles, each guided by a dedicated update strategy. A sliding-window-based competition mechanism is introduced to evaluate recent evolutionary efficiency and to drive adaptive particle migration between subpopulations. Through this biomimetic competition and migration process, the population structure can be dynamically reorganized in response to the current search state while preserving the continuity of particle trajectories. Extensive experimental results on the CEC2017 benchmark suite demonstrate that DCPSO achieves competitive performance compared with the canonical PSO and several representative state-of-the-art variants. Additional experiments on engineering design problems further confirm the robustness and practical effectiveness of the proposed framework.

Beyond benchmark validation, the proposed framework may be applicable to large-scale engineering design, high-dimensional parameter tuning, and data-driven optimization tasks where adaptive resource allocation and structural flexibility are beneficial. The competition-driven mechanism could also be explored in scenarios involving complex multimodal landscapes or evolving search demands.

Future research may investigate extensions to multiobjective, constrained, and dynamic optimization problems. Exploring more flexible multi-subpopulation frameworks with heterogeneous search behaviors and adaptively adjustable sizes may further enhance scalability and generalization ability. Incorporating adaptive or self-tuning parameter control strategies could improve robustness and reduce manual configuration effort. In addition, formal convergence analysis or probabilistic modeling of the competition and migration dynamics may provide deeper theoretical understanding of the proposed mechanism. These directions may further strengthen the applicability and theoretical foundation of the framework.

## Figures and Tables

**Table 1:** Parameter settings of DCPSO and the peer algorithms.

Algorithm	Other Parameters
DCPSO	$c_{1}=c_{2}=1.5$, $c_{3}=0.1$, W=10, K=3
ACEPSO [34]	$D_{p}=0.7$, $D_{is}=0.65$, $N_{ne}=2.4$
AFMPSO [42]	ε=0.01·D∖N,r=0.5
VPPSO [43]	α=0.3, $N_{1}=50$, $N_{2}=50$
SDPSO [27]	α=0.1, r=4, LP=200
MPSO [18]	r(t+1)=4r(t)(1−r(t)), r(0)=rand
PSO [11]	$c_{1}=c_{2}=1.5$

**Table 2:** Experimental results on the CEC2017 benchmark functions.

Func.	DCPSO	ACEPSO	AFMPSO	VPPSO	SDPSO	MPSO	PSO
F1	$1.197\times 10^{3}$	$3.762\times 10^{3}$	$9.568\times 10^{3}$	$2.558\times 10^{3}$	$6.429\times 10^{3}$	$1.798\times 10^{8}$	$1.263\times 10^{10}$
	$9.667\times 10^{2}$	$1.368\times 10^{3}$	$4.426\times 10^{3}$	$4.619\times 10^{3}$	$6.363\times 10^{3}$	$2.687\times 10^{8}$	$6.178\times 10^{9}$
	1	3	5	2	4	6	7
F3	$7.418\times 10^{3}$	$8.946\times 10^{2}$	$9.952\times 10^{1}$	$1.646\times 10^{3}$	$2.729\times 10^{4}$	$8.249\times 10^{3}$	$2.444\times 10^{4}$
	$2.261\times 10^{3}$	$1.285\times 10^{3}$	$4.419\times 10^{1}$	$6.942\times 10^{2}$	$7.283\times 10^{3}$	$3.305\times 10^{3}$	$1.678\times 10^{4}$
	4	2	1	3	7	5	6
F4	$4.988\times 10^{0}$	$9.536\times 10^{1}$	$8.425\times 10^{1}$	$9.407\times 10^{1}$	$8.788\times 10^{1}$	$1.205\times 10^{2}$	$1.322\times 10^{3}$
	$9.325\times 10^{0}$	$1.134\times 10^{1}$	$2.243\times 10^{1}$	$2.072\times 10^{1}$	$1.031\times 10^{1}$	$5.031\times 10^{1}$	$1.222\times 10^{3}$
	1	5	2	4	3	6	7
F5	$6.419\times 10^{1}$	$8.776\times 10^{1}$	$1.216\times 10^{2}$	$1.601\times 10^{2}$	$8.612\times 10^{1}$	$7.107\times 10^{1}$	$1.458\times 10^{2}$
	$1.160\times 10^{1}$	$3.906\times 10^{0}$	$1.662\times 10^{1}$	$2.200\times 10^{1}$	$6.566\times 10^{1}$	$2.516\times 10^{1}$	$3.895\times 10^{1}$
	1	4	5	7	3	2	6
F6	$2.317\times 10^{-4}$	$6.461\times 10^{0}$	$3.815\times 10^{1}$	$4.180\times 10^{1}$	$2.098\times 10^{-1}$	$3.662\times 10^{0}$	$1.955\times 10^{1}$
	$3.377\times 10^{-4}$	$5.575\times 10^{-1}$	$6.309\times 10^{0}$	$7.208\times 10^{0}$	$1.940\times 10^{-1}$	$1.760\times 10^{0}$	$7.027\times 10^{0}$
	1	4	6	7	2	3	5
F7	$8.527\times 10^{1}$	$6.009\times 10^{1}$	$1.786\times 10^{2}$	$2.455\times 10^{2}$	$1.426\times 10^{2}$	$1.313\times 10^{2}$	$2.371\times 10^{2}$
	$1.008\times 10^{1}$	$6.067\times 10^{0}$	$3.200\times 10^{1}$	$4.535\times 10^{1}$	$7.277\times 10^{1}$	$3.650\times 10^{1}$	$6.369\times 10^{1}$
	2	1	5	7	4	3	6
F8	$6.023\times 10^{1}$	$8.484\times 10^{1}$	$9.237\times 10^{1}$	$1.197\times 10^{2}$	$5.298\times 10^{1}$	$6.851\times 10^{1}$	$1.421\times 10^{2}$
	$1.110\times 10^{1}$	$3.998\times 10^{0}$	$1.615\times 10^{1}$	$2.164\times 10^{1}$	$4.207\times 10^{1}$	$2.591\times 10^{1}$	$3.414\times 10^{1}$
	2	4	5	6	1	3	7
F9	$2.218\times 10^{0}$	$3.613\times 10^{1}$	$1.524\times 10^{3}$	$2.310\times 10^{3}$	$1.213\times 10^{1}$	$1.217\times 10^{2}$	$2.566\times 10^{3}$
	$1.758\times 10^{0}$	$1.221\times 10^{1}$	$4.900\times 10^{2}$	$7.052\times 10^{2}$	$1.343\times 10^{1}$	$1.259\times 10^{2}$	$1.221\times 10^{3}$
	1	3	5	6	2	4	7
F10	$3.443\times 10^{3}$	$3.135\times 10^{3}$	$3.929\times 10^{3}$	$3.821\times 10^{3}$	$7.155\times 10^{3}$	$4.223\times 10^{3}$	$4.058\times 10^{3}$
	$6.198\times 10^{2}$	$2.040\times 10^{3}$	$5.552\times 10^{2}$	$4.113\times 10^{2}$	$2.378\times 10^{2}$	$6.733\times 10^{2}$	$6.566\times 10^{2}$
	2	1	4	3	7	6	5
F11	$7.935\times 10^{1}$	$1.066\times 10^{2}$	$1.496\times 10^{2}$	$1.303\times 10^{2}$	$1.108\times 10^{2}$	$1.212\times 10^{2}$	$5.463\times 10^{2}$
	$1.707\times 10^{1}$	$5.754\times 10^{1}$	$3.997\times 10^{1}$	$5.111\times 10^{1}$	$5.543\times 10^{1}$	$5.544\times 10^{1}$	$3.068\times 10^{2}$
	1	2	6	5	3	4	7
F12	$1.487\times 10^{4}$	$5.091\times 10^{5}$	$1.659\times 10^{6}$	$2.715\times 10^{6}$	$2.184\times 10^{5}$	$1.895\times 10^{6}$	$5.703\times 10^{8}$
	$6.154\times 10^{3}$	$2.499\times 10^{4}$	$1.162\times 10^{6}$	$1.837\times 10^{6}$	$4.046\times 10^{5}$	$1.866\times 10^{6}$	$5.340\times 10^{8}$
	1	2	4	6	3	5	7
F13	$5.633\times 10^{3}$	$1.397\times 10^{4}$	$3.686\times 10^{4}$	$7.255\times 10^{4}$	$3.033\times 10^{4}$	$1.399\times 10^{4}$	$7.601\times 10^{7}$
	$4.118\times 10^{3}$	$1.197\times 10^{4}$	$1.542\times 10^{4}$	$3.865\times 10^{4}$	$2.477\times 10^{4}$	$8.522\times 10^{3}$	$3.258\times 10^{8}$
	1	2	5	6	4	3	7
F14	$2.195\times 10^{3}$	$5.868\times 10^{3}$	$3.830\times 10^{2}$	$1.499\times 10^{3}$	$2.998\times 10^{4}$	$2.735\times 10^{3}$	$8.627\times 10^{4}$
	$1.749\times 10^{3}$	$2.578\times 10^{3}$	$1.782\times 10^{2}$	$1.636\times 10^{3}$	$2.568\times 10^{4}$	$2.692\times 10^{3}$	$8.161\times 10^{4}$
	3	5	1	2	6	4	7
F15	$1.373\times 10^{3}$	$1.871\times 10^{3}$	$8.951\times 10^{3}$	$3.932\times 10^{4}$	$7.638\times 10^{3}$	$1.558\times 10^{3}$	$8.265\times 10^{4}$
	$1.099\times 10^{3}$	$1.249\times 10^{3}$	$5.169\times 10^{3}$	$2.108\times 10^{4}$	$8.859\times 10^{3}$	$1.369\times 10^{3}$	$7.095\times 10^{4}$
	1	3	5	6	4	2	7
F16	$5.423\times 10^{2}$	$6.697\times 10^{2}$	$1.142\times 10^{3}$	$1.135\times 10^{3}$	$9.480\times 10^{2}$	$7.791\times 10^{2}$	$1.261\times 10^{3}$
	$1.484\times 10^{2}$	$2.449\times 10^{2}$	$2.770\times 10^{2}$	$3.162\times 10^{2}$	$4.574\times 10^{2}$	$2.949\times 10^{2}$	$3.170\times 10^{2}$
	1	2	6	5	4	3	7
F17	$1.329\times 10^{2}$	$2.540\times 10^{2}$	$5.753\times 10^{2}$	$5.080\times 10^{2}$	$2.175\times 10^{2}$	$3.102\times 10^{2}$	$6.496\times 10^{2}$
	$5.634\times 10^{1}$	$5.125\times 10^{1}$	$1.736\times 10^{2}$	$1.862\times 10^{2}$	$1.300\times 10^{2}$	$1.531\times 10^{2}$	$2.267\times 10^{2}$
	1	3	6	5	2	4	7
F18	$6.136\times 10^{4}$	$1.435\times 10^{5}$	$3.914\times 10^{4}$	$7.143\times 10^{4}$	$5.970\times 10^{5}$	$8.640\times 10^{4}$	$9.791\times 10^{5}$
	$2.614\times 10^{4}$	$4.023\times 10^{4}$	$1.656\times 10^{4}$	$2.817\times 10^{4}$	$5.960\times 10^{5}$	$8.542\times 10^{4}$	$2.213\times 10^{6}$
	2	5	1	3	6	4	7
F19	$2.660\times 10^{3}$	$5.309\times 10^{3}$	$5.970\times 10^{3}$	$2.612\times 10^{4}$	$1.054\times 10^{4}$	$3.315\times 10^{3}$	$2.709\times 10^{6}$
	$1.988\times 10^{3}$	$1.465\times 10^{3}$	$5.370\times 10^{3}$	$2.117\times 10^{4}$	$1.132\times 10^{4}$	$2.800\times 10^{3}$	$4.869\times 10^{6}$
	1	3	4	6	5	2	7
F20	$2.623\times 10^{2}$	$3.424\times 10^{2}$	$5.209\times 10^{2}$	$5.121\times 10^{2}$	$2.348\times 10^{2}$	$2.245\times 10^{2}$	$4.501\times 10^{2}$
	$4.629\times 10^{1}$	$4.700\times 10^{1}$	$1.230\times 10^{2}$	$1.256\times 10^{2}$	$1.396\times 10^{2}$	$1.080\times 10^{2}$	$1.902\times 10^{2}$
	3	4	7	6	2	1	5
F21	$2.541\times 10^{2}$	$2.875\times 10^{2}$	$3.120\times 10^{2}$	$3.539\times 10^{2}$	$2.692\times 10^{2}$	$2.622\times 10^{2}$	$3.526\times 10^{2}$
	$1.021\times 10^{1}$	$4.859\times 10^{0}$	$1.801\times 10^{1}$	$5.407\times 10^{1}$	$4.292\times 10^{1}$	$1.878\times 10^{1}$	$4.136\times 10^{1}$
	1	4	5	7	3	2	6
F22	$1.000\times 10^{2}$	$2.279\times 10^{2}$	$8.914\times 10^{2}$	$1.959\times 10^{3}$	$2.607\times 10^{3}$	$3.618\times 10^{2}$	$3.152\times 10^{3}$
	$2.215\times 10^{-13}$	$1.684\times 10^{3}$	$1.515\times 10^{3}$	$1.930\times 10^{3}$	$3.354\times 10^{3}$	$8.871\times 10^{2}$	$1.661\times 10^{3}$
	1	2	4	5	6	3	7
F23	$4.141\times 10^{2}$	$4.801\times 10^{2}$	$6.553\times 10^{2}$	$6.042\times 10^{2}$	$4.273\times 10^{2}$	$4.341\times 10^{2}$	$6.520\times 10^{2}$
	$2.829\times 10^{1}$	$1.875\times 10^{1}$	$6.660\times 10^{1}$	$6.131\times 10^{1}$	$1.787\times 10^{1}$	$2.852\times 10^{1}$	$7.561\times 10^{1}$
	1	4	7	5	2	3	6
F24	$4.768\times 10^{2}$	$5.930\times 10^{2}$	$6.965\times 10^{2}$	$6.588\times 10^{2}$	$5.364\times 10^{2}$	$4.901\times 10^{2}$	$7.336\times 10^{2}$
	$1.170\times 10^{1}$	$1.330\times 10^{1}$	$6.009\times 10^{1}$	$5.936\times 10^{1}$	$5.028\times 10^{1}$	$2.579\times 10^{1}$	$7.378\times 10^{1}$
	1	4	6	5	3	2	7
F25	$3.873\times 10^{2}$	$4.008\times 10^{2}$	$4.146\times 10^{2}$	$4.071\times 10^{2}$	$3.880\times 10^{2}$	$4.669\times 10^{2}$	$6.265\times 10^{2}$
	$1.252\times 10^{0}$	$1.634\times 10^{0}$	$2.237\times 10^{1}$	$2.092\times 10^{1}$	$1.238\times 10^{0}$	$3.344\times 10^{1}$	$2.116\times 10^{2}$
	1	3	5	4	2	6	7
F26	$9.212\times 10^{2}$	$4.472\times 10^{2}$	$3.264\times 10^{3}$	$2.669\times 10^{3}$	$1.683\times 10^{3}$	$1.415\times 10^{3}$	$3.512\times 10^{3}$
	$7.164\times 10^{2}$	$4.066\times 10^{2}$	$1.256\times 10^{3}$	$1.285\times 10^{3}$	$1.982\times 10^{2}$	$1.001\times 10^{3}$	$6.532\times 10^{2}$
	2	1	6	5	4	3	7
F27	$5.232\times 10^{2}$	$5.416\times 10^{2}$	$6.099\times 10^{2}$	$6.153\times 10^{2}$	$5.296\times 10^{2}$	$5.457\times 10^{2}$	$6.221\times 10^{2}$
	$7.246\times 10^{0}$	$1.475\times 10^{1}$	$4.502\times 10^{1}$	$6.734\times 10^{1}$	$1.541\times 10^{1}$	$2.282\times 10^{1}$	$6.306\times 10^{1}$
	1	3	5	6	2	4	7
F28	$3.000\times 10^{2}$	$4.296\times 10^{2}$	$4.301\times 10^{2}$	$4.292\times 10^{2}$	$4.459\times 10^{2}$	$5.838\times 10^{2}$	$1.616\times 10^{3}$
	$2.163\times 10^{-6}$	$3.987\times 10^{1}$	$1.830\times 10^{1}$	$2.133\times 10^{1}$	$5.399\times 10^{1}$	$9.307\times 10^{1}$	$9.161\times 10^{2}$
	1	3	4	2	5	6	7
F29	$6.988\times 10^{2}$	$7.366\times 10^{2}$	$1.425\times 10^{3}$	$1.421\times 10^{3}$	$6.376\times 10^{2}$	$7.655\times 10^{2}$	$1.197\times 10^{3}$
	$6.947\times 10^{1}$	$1.314\times 10^{2}$	$2.278\times 10^{2}$	$2.101\times 10^{2}$	$1.267\times 10^{2}$	$2.090\times 10^{2}$	$2.809\times 10^{2}$
	2	3	7	6	1	4	5
F30	$3.383\times 10^{3}$	$1.137\times 10^{4}$	$1.616\times 10^{5}$	$5.854\times 10^{5}$	$8.408\times 10^{3}$	$1.751\times 10^{4}$	$5.346\times 10^{6}$
	$5.944\times 10^{2}$	$1.827\times 10^{3}$	$7.144\times 10^{4}$	$2.515\times 10^{5}$	$4.069\times 10^{3}$	$2.235\times 10^{4}$	$7.217\times 10^{6}$
	1	3	4	6	2	5	7
Mean Rank	1.448	3.034	4.690	5.034	3.517	3.724	6.552
Final Rank	1	2	5	6	3	4	7

**Table 3:** Wilcoxon signed-rank test results of DCPSO against 6 algorithms on the CEC2017 benchmark functions with significance level α=0.05.

Func.	ACEPSO	AFMPSO	VPPSO	SDPSO	MPSO	PSO
F1	+	+	+	+	+	+
F3	−	−	−	+	≈	+
F4	+	+	+	+	+	+
F5	+	+	+	+	+	+
F6	+	+	+	+	+	+
F7	−	+	+	+	+	+
F8	+	+	+	+	≈	+
F9	+	+	+	+	+	+
F10	−	+	+	+	+	+
F11	+	+	+	+	+	+
F12	+	+	+	+	+	+
F13	+	+	+	+	+	+
F14	+	≈	-	+	+	+
F15	+	+	+	+	+	+
F16	+	+	+	+	+	+
F17	+	+	+	+	+	+
F18	+	−	+	+	+	+
F19	+	+	+	+	+	+
F20	+	+	+	≈	≈	+
F21	+	+	+	≈	+	+
F22	+	+	+	+	+	+
F23	+	+	+	−	+	+
F24	+	+	+	+	+	+
F25	+	+	+	+	+	+
F26	−	+	+	+	+	+
F27	+	+	+	+	+	+
F28	+	+	+	+	+	+
F29	+	+	+	−	−	+
F30	+	+	+	+	+	+
Better	25	26	27	25	25	29
Similar	0	1	0	2	3	0
Worse	4	2	2	2	1	0

**Table 4:** Sensitivity analysis of parameter *W* on the CEC2017 benchmark functions. Reported values are the mean errors over 30 independent runs.

Func.	W=1	W=5	W=10	W=15	W=20
F1	$1.747\times 10^{3}$	$1.236\times 10^{3}$	$1.115\times 10^{3}$	$1.095\times 10^{3}$	$1.438\times 10^{3}$
F3	$7.490\times 10^{3}$	$7.670\times 10^{3}$	$7.724\times 10^{3}$	$7.776\times 10^{3}$	$8.446\times 10^{3}$
F4	$2.771\times 10^{1}$	$6.532\times 10^{0}$	$4.091\times 10^{0}$	$4.418\times 10^{0}$	$6.521\times 10^{0}$
F5	$7.184\times 10^{1}$	$6.585\times 10^{1}$	$6.537\times 10^{1}$	$6.766\times 10^{1}$	$6.615\times 10^{1}$
F6	$4.172\times 10^{-4}$	$1.806\times 10^{-4}$	$1.783\times 10^{-4}$	$2.559\times 10^{-4}$	$2.878\times 10^{-4}$
F7	$8.483\times 10^{1}$	$7.975\times 10^{1}$	$8.131\times 10^{1}$	$8.467\times 10^{1}$	$8.482\times 10^{1}$
F8	$6.609\times 10^{1}$	$6.390\times 10^{1}$	$6.195\times 10^{1}$	$6.282\times 10^{1}$	$6.526\times 10^{1}$
F9	$3.143\times 10^{0}$	$1.981\times 10^{0}$	$1.655\times 10^{0}$	$2.730\times 10^{0}$	$2.780\times 10^{0}$
F10	$3.917\times 10^{3}$	$3.414\times 10^{3}$	$3.504\times 10^{3}$	$3.472\times 10^{3}$	$3.573\times 10^{3}$
F11	$8.592\times 10^{1}$	$7.932\times 10^{1}$	$7.383\times 10^{1}$	$8.099\times 10^{1}$	$8.233\times 10^{1}$
F12	$2.568\times 10^{4}$	$1.724\times 10^{4}$	$1.618\times 10^{4}$	$1.811\times 10^{4}$	$1.933\times 10^{4}$
F13	$8.744\times 10^{3}$	$6.194\times 10^{3}$	$6.139\times 10^{3}$	$5.479\times 10^{3}$	$5.504\times 10^{3}$
F14	$1.662\times 10^{3}$	$2.650\times 10^{3}$	$2.523\times 10^{3}$	$2.627\times 10^{3}$	$2.692\times 10^{3}$
F15	$1.841\times 10^{3}$	$9.469\times 10^{2}$	$8.552\times 10^{2}$	$1.206\times 10^{3}$	$1.222\times 10^{3}$
F16	$5.225\times 10^{2}$	$5.688\times 10^{2}$	$5.272\times 10^{2}$	$5.428\times 10^{2}$	$5.091\times 10^{2}$
F17	$1.570\times 10^{2}$	$1.287\times 10^{2}$	$1.194\times 10^{2}$	$1.229\times 10^{2}$	$1.425\times 10^{2}$
F18	$7.338\times 10^{4}$	$6.425\times 10^{4}$	$6.730\times 10^{4}$	$7.060\times 10^{4}$	$7.012\times 10^{4}$
F19	$2.877\times 10^{3}$	$2.701\times 10^{3}$	$2.238\times 10^{3}$	$2.075\times 10^{3}$	$2.155\times 10^{3}$
F20	$2.718\times 10^{2}$	$2.696\times 10^{2}$	$2.630\times 10^{2}$	$2.637\times 10^{2}$	$2.660\times 10^{2}$
F21	$2.629\times 10^{2}$	$2.588\times 10^{2}$	$2.550\times 10^{2}$	$2.579\times 10^{2}$	$2.556\times 10^{2}$
F22	$1.000\times 10^{2}$	$1.000\times 10^{2}$	$1.000\times 10^{2}$	$1.000\times 10^{2}$	$1.000\times 10^{2}$
F23	$4.193\times 10^{2}$	$4.157\times 10^{2}$	$4.143\times 10^{2}$	$4.108\times 10^{2}$	$4.148\times 10^{2}$
F24	$4.748\times 10^{2}$	$4.756\times 10^{2}$	$4.750\times 10^{2}$	$4.729\times 10^{2}$	$4.734\times 10^{2}$
F25	$3.867\times 10^{2}$	$3.869\times 10^{2}$	$3.869\times 10^{2}$	$3.872\times 10^{2}$	$3.878\times 10^{2}$
F26	$1.049\times 10^{3}$	$7.221\times 10^{2}$	$7.666\times 10^{2}$	$8.333\times 10^{2}$	$8.464\times 10^{2}$
F27	$5.228\times 10^{2}$	$5.195\times 10^{2}$	$5.199\times 10^{2}$	$5.211\times 10^{2}$	$5.209\times 10^{2}$
F28	$3.137\times 10^{2}$	$3.000\times 10^{2}$	$3.000\times 10^{2}$	$3.000\times 10^{2}$	$3.014\times 10^{2}$
F29	$7.187\times 10^{2}$	$6.831\times 10^{2}$	$6.727\times 10^{2}$	$6.796\times 10^{2}$	$7.090\times 10^{2}$
F30	$3.922\times 10^{3}$	$3.636\times 10^{3}$	$3.418\times 10^{3}$	$3.492\times 10^{3}$	$3.643\times 10^{3}$
Mean Rank	4.41	2.79	1.79	2.62	3.38
Final Rank	5	3	1	2	4

**Table 5:** Sensitivity analysis of parameter *k* on the CEC2017 benchmark functions. Reported values are the mean errors over 30 independent runs.

Func.	k=1	k=2	k=3	k=4	k=5
F1	$9.712\times 10^{2}$	$1.115\times 10^{3}$	$1.187\times 10^{3}$	$1.184\times 10^{3}$	$1.147\times 10^{3}$
F3	$1.375\times 10^{4}$	$8.998\times 10^{3}$	$7.421\times 10^{3}$	$6.069\times 10^{3}$	$5.460\times 10^{3}$
F4	$2.023\times 10^{1}$	$7.371\times 10^{0}$	$4.478\times 10^{0}$	$1.302\times 10^{1}$	$7.252\times 10^{0}$
F5	$8.064\times 10^{1}$	$6.754\times 10^{1}$	$6.669\times 10^{1}$	$6.494\times 10^{1}$	$6.498\times 10^{1}$
F6	$7.179\times 10^{-2}$	$4.672\times 10^{-4}$	$2.373\times 10^{-4}$	$4.937\times 10^{-4}$	$1.008\times 10^{-3}$
F7	$9.829\times 10^{1}$	$8.784\times 10^{1}$	$8.306\times 10^{1}$	$8.175\times 10^{1}$	$8.169\times 10^{1}$
F8	$7.189\times 10^{1}$	$6.781\times 10^{1}$	$6.313\times 10^{1}$	$6.201\times 10^{1}$	$6.113\times 10^{1}$
F9	$3.180\times 10^{1}$	$4.248\times 10^{0}$	$2.218\times 10^{0}$	$3.084\times 10^{0}$	$2.849\times 10^{0}$
F10	$3.955\times 10^{3}$	$3.521\times 10^{3}$	$3.534\times 10^{3}$	$3.393\times 10^{3}$	$3.513\times 10^{3}$
F11	$8.947\times 10^{1}$	$8.383\times 10^{1}$	$8.265\times 10^{1}$	$8.119\times 10^{1}$	$7.951\times 10^{1}$
F12	$2.361\times 10^{4}$	$1.975\times 10^{4}$	$1.826\times 10^{4}$	$1.815\times 10^{4}$	$1.979\times 10^{4}$
F13	$7.288\times 10^{3}$	$5.365\times 10^{3}$	$5.678\times 10^{3}$	$5.793\times 10^{3}$	$5.571\times 10^{3}$
F14	$2.128\times 10^{3}$	$2.120\times 10^{3}$	$2.413\times 10^{3}$	$2.886\times 10^{3}$	$3.338\times 10^{3}$
F15	$1.260\times 10^{3}$	$1.375\times 10^{3}$	$1.228\times 10^{3}$	$1.044\times 10^{3}$	$1.569\times 10^{3}$
F16	$5.915\times 10^{2}$	$5.842\times 10^{2}$	$5.555\times 10^{2}$	$5.201\times 10^{2}$	$5.696\times 10^{2}$
F17	$1.481\times 10^{2}$	$1.418\times 10^{2}$	$1.353\times 10^{2}$	$1.439\times 10^{2}$	$1.423\times 10^{2}$
F18	$6.047\times 10^{4}$	$6.165\times 10^{4}$	$6.620\times 10^{4}$	$7.481\times 10^{4}$	$7.034\times 10^{4}$
F19	$2.648\times 10^{3}$	$2.048\times 10^{3}$	$2.660\times 10^{3}$	$2.344\times 10^{3}$	$2.523\times 10^{3}$
F20	$2.712\times 10^{2}$	$2.715\times 10^{2}$	$2.623\times 10^{2}$	$2.702\times 10^{2}$	$2.664\times 10^{2}$
F21	$2.684\times 10^{2}$	$2.571\times 10^{2}$	$2.573\times 10^{2}$	$2.563\times 10^{2}$	$2.533\times 10^{2}$
F22	$1.000\times 10^{2}$	$1.000\times 10^{2}$	$1.000\times 10^{2}$	$1.000\times 10^{2}$	$1.000\times 10^{2}$
F23	$4.290\times 10^{2}$	$4.188\times 10^{2}$	$4.110\times 10^{2}$	$4.161\times 10^{2}$	$4.138\times 10^{2}$
F24	$4.887\times 10^{2}$	$4.826\times 10^{2}$	$4.750\times 10^{2}$	$4.752\times 10^{2}$	$4.760\times 10^{2}$
F25	$3.870\times 10^{2}$	$3.874\times 10^{2}$	$3.869\times 10^{2}$	$3.868\times 10^{2}$	$3.866\times 10^{2}$
F26	$1.166\times 10^{3}$	$9.869\times 10^{2}$	$8.867\times 10^{2}$	$8.259\times 10^{2}$	$8.591\times 10^{2}$
F27	$5.246\times 10^{2}$	$5.226\times 10^{2}$	$5.216\times 10^{2}$	$5.223\times 10^{2}$	$5.211\times 10^{2}$
F28	$3.006\times 10^{2}$	$3.000\times 10^{2}$	$3.000\times 10^{2}$	$3.000\times 10^{2}$	$3.000\times 10^{2}$
F29	$7.875\times 10^{2}$	$7.039\times 10^{2}$	$6.928\times 10^{2}$	$6.629\times 10^{2}$	$7.042\times 10^{2}$
F30	$3.593\times 10^{3}$	$3.412\times 10^{3}$	$3.769\times 10^{3}$	$3.681\times 10^{3}$	$3.693\times 10^{3}$
Mean Rank	4.38	3.24	2.34	2.41	2.62
Final Rank	5	4	1	2	3

**Table 6:** Ablation study results of DCPSO and its variants on the CEC2017 functions.

Func.	DCPSO	DCPSO-E	DCPSO-X	DCPSO-DC	PSO
F1	$1.197\times 10^{3}$	$5.052\times 10^{5}$	$3.073\times 10^{3}$	$2.718\times 10^{3}$	$9.078\times 10^{9}$
F3	$7.418\times 10^{3}$	$6.368\times 10^{4}$	$1.086\times 10^{4}$	$2.728\times 10^{4}$	$2.606\times 10^{4}$
F4	$4.988\times 10^{0}$	$1.338\times 10^{2}$	$2.881\times 10^{1}$	$8.094\times 10^{1}$	$1.175\times 10^{3}$
F5	$6.419\times 10^{1}$	$1.539\times 10^{2}$	$7.412\times 10^{1}$	$8.850\times 10^{1}$	$1.402\times 10^{2}$
F6	$2.317\times 10^{-4}$	$2.622\times 10^{0}$	$1.091\times 10^{-3}$	$1.403\times 10^{-3}$	$1.571\times 10^{1}$
F7	$8.527\times 10^{1}$	$2.136\times 10^{2}$	$8.885\times 10^{1}$	$1.007\times 10^{2}$	$2.063\times 10^{2}$
F8	$6.023\times 10^{1}$	$1.549\times 10^{2}$	$7.177\times 10^{1}$	$9.197\times 10^{1}$	$1.343\times 10^{2}$
F9	$2.218\times 10^{0}$	$8.018\times 10^{2}$	$3.175\times 10^{0}$	$9.102\times 10^{0}$	$2.567\times 10^{3}$
F10	$3.443\times 10^{3}$	$5.974\times 10^{3}$	$3.312\times 10^{3}$	$4.032\times 10^{3}$	$3.916\times 10^{3}$
F11	$7.935\times 10^{1}$	$1.947\times 10^{2}$	$1.031\times 10^{2}$	$1.285\times 10^{2}$	$3.945\times 10^{2}$
F12	$1.487\times 10^{4}$	$2.929\times 10^{6}$	$2.655\times 10^{4}$	$1.094\times 10^{5}$	$4.791\times 10^{8}$
F13	$5.633\times 10^{3}$	$1.533\times 10^{5}$	$1.174\times 10^{4}$	$1.239\times 10^{4}$	$6.902\times 10^{6}$
F14	$2.195\times 10^{3}$	$1.393\times 10^{4}$	$3.355\times 10^{3}$	$5.619\times 10^{3}$	$3.965\times 10^{4}$
F15	$1.373\times 10^{3}$	$2.484\times 10^{4}$	$3.432\times 10^{3}$	$3.805\times 10^{3}$	$6.744\times 10^{4}$
F16	$5.423\times 10^{2}$	$8.786\times 10^{2}$	$5.792\times 10^{2}$	$6.760\times 10^{2}$	$1.137\times 10^{3}$
F17	$1.329\times 10^{2}$	$2.185\times 10^{2}$	$1.859\times 10^{2}$	$1.506\times 10^{2}$	$6.010\times 10^{2}$
F18	$6.136\times 10^{4}$	$3.746\times 10^{5}$	$9.743\times 10^{4}$	$1.797\times 10^{5}$	$2.851\times 10^{5}$
F19	$2.660\times 10^{3}$	$2.373\times 10^{4}$	$5.036\times 10^{3}$	$5.105\times 10^{3}$	$2.192\times 10^{6}$
F20	$2.623\times 10^{2}$	$3.166\times 10^{2}$	$2.893\times 10^{2}$	$2.633\times 10^{2}$	$3.941\times 10^{2}$
F21	$2.541\times 10^{2}$	$3.147\times 10^{2}$	$2.675\times 10^{2}$	$2.788\times 10^{2}$	$3.602\times 10^{2}$
F22	$1.000\times 10^{2}$	$2.720\times 10^{2}$	$1.000\times 10^{2}$	$1.007\times 10^{2}$	$2.892\times 10^{3}$
F23	$4.141\times 10^{2}$	$4.865\times 10^{2}$	$4.253\times 10^{2}$	$4.347\times 10^{2}$	$6.163\times 10^{2}$
F24	$4.768\times 10^{2}$	$5.560\times 10^{2}$	$4.790\times 10^{2}$	$4.952\times 10^{2}$	$7.065\times 10^{2}$
F25	$3.873\times 10^{2}$	$3.918\times 10^{2}$	$3.877\times 10^{2}$	$3.955\times 10^{2}$	$6.105\times 10^{2}$
F26	$9.212\times 10^{2}$	$1.382\times 10^{3}$	$1.218\times 10^{3}$	$1.173\times 10^{3}$	$3.534\times 10^{3}$
F27	$5.232\times 10^{2}$	$5.389\times 10^{2}$	$5.293\times 10^{2}$	$5.220\times 10^{2}$	$6.025\times 10^{2}$
F28	$3.000\times 10^{2}$	$4.629\times 10^{2}$	$3.208\times 10^{2}$	$3.818\times 10^{2}$	$1.153\times 10^{3}$
F29	$6.988\times 10^{2}$	$9.078\times 10^{2}$	$7.700\times 10^{2}$	$7.482\times 10^{2}$	$1.210\times 10^{3}$
F30	$3.383\times 10^{3}$	$3.132\times 10^{5}$	$4.515\times 10^{3}$	$4.819\times 10^{3}$	$2.721\times 10^{6}$

**Table 7:** Comparison results on the three-bar truss design problem.

Algorithm	Optimized Result			Optimization Variable	
	Best	Mean	Std.	$x_{1}$	$x_{2}$
DCPSO	$2.63896\times 10^{2}$	$2.63896\times 10^{2}$	$1.33666\times 10^{-5}$	0.7887	0.4083
ACEPSO	$2.63896\times 10^{2}$	$2.63896\times 10^{2}$	$1.56130\times 10^{-5}$	0.7887	0.4082
AFMPSO	$2.63896\times 10^{2}$	$2.63898\times 10^{2}$	$4.27073\times 10^{-3}$	0.7887	0.4083
VPPSO	$2.63896\times 10^{2}$	$2.63896\times 10^{2}$	$4.48355\times 10^{-4}$	0.7887	0.4083
SDPSO	$2.63896\times 10^{2}$	$2.63896\times 10^{2}$	$2.50774\times 10^{-5}$	0.7887	0.4082
MPSO	$2.63896\times 10^{2}$	$2.63896\times 10^{2}$	$4.01519\times 10^{-6}$	0.7887	0.4082
PSO	$2.63896\times 10^{2}$	$2.63896\times 10^{2}$	$2.50289\times 10^{-5}$	0.7887	0.4082

**Table 8:** Comparison results on the gear train design problem.

Algorithm	Optimized Result			Optimization Variable			
	Best	Mean	Std.	$x_{1}$	$x_{2}$	$x_{3}$	$x_{4}$
DCPSO	$2.70086\times 10^{-12}$	$6.09707\times 10^{-12}$	$7.72399\times 10^{-12}$	49.4702	15.8649	19.0769	43.2132
ACEPSO	$2.70086\times 10^{-12}$	$4.47249\times 10^{-10}$	$4.76498\times 10^{-10}$	48.8431	15.8243	19.0019	43.0089
AFMPSO	$2.70086\times 10^{-12}$	$5.02722\times 10^{-10}$	$5.97481\times 10^{-10}$	42.5040	15.9593	19.2789	48.9835
VPPSO	$2.70086\times 10^{-12}$	$5.58935\times 10^{-10}$	$6.75837\times 10^{-10}$	48.6641	19.1968	16.4948	43.4540
SDPSO	$2.70086\times 10^{-12}$	$1.08338\times 10^{-10}$	$2.76852\times 10^{-10}$	48.5168	19.1123	15.6856	43.1325
MPSO	$1.36165\times 10^{-9}$	$1.37192\times 10^{-8}$	$1.29235\times 10^{-8}$	60.0000	33.7594	14.1274	54.9405
PSO	$2.70086\times 10^{-12}$	$1.70802\times 10^{-9}$	$4.85272\times 10^{-9}$	48.5989	19.3534	16.1911	42.9620

**Table 9:** Comparison results on the pressure vessel design problem.

Algorithm	Optimized Result			Optimization Variable			
	Best	Mean	Std.	$x_{1}$	$x_{2}$	$x_{3}$	$x_{4}$
DCPSO	$6.05971\times 10^{3}$	$6.17872\times 10^{3}$	$1.34649\times 10^{2}$	13.2808	7.0891	42.0984	176.6366
ACEPSO	$6.05971\times 10^{3}$	$6.42932\times 10^{3}$	$3.02489\times 10^{2}$	12.9102	7.1233	42.0984	176.6366
AFMPSO	$6.05972\times 10^{3}$	$6.45425\times 10^{3}$	$2.26756\times 10^{2}$	13.4097	6.5622	42.0984	176.6368
VPPSO	$6.05971\times 10^{3}$	$6.52902\times 10^{3}$	$3.52286\times 10^{2}$	12.5377	7.1640	42.0984	176.6366
SDPSO	$6.05971\times 10^{3}$	$6.06619\times 10^{3}$	$1.24940\times 10^{1}$	12.8702	7.1830	42.0984	176.6366
MPSO	$6.09053\times 10^{3}$	$6.53380\times 10^{3}$	$3.39194\times 10^{2}$	13.9664	7.0662	45.3368	140.2538
PSO	$6.05971\times 10^{3}$	$6.20891\times 10^{3}$	$3.44106\times 10^{2}$	12.5671	7.2428	42.0984	176.6366

## Data Availability

The original contributions presented in this study are contained in this paper. Further inquiries can be directed to the corresponding author.

## Abbreviations

The following abbreviations are used in this manuscript:

ACEPSO	Adaptive Co-Evolved Particle Swarm Optimization
AFMPSO	Adaptive Forgetting Multi-Strategy Particle Swarm Optimization
DCPSO	Dual-Subpopulation Competitive Particle Swarm Optimization
MPSO	Modified Particle Swarm Optimization
PSO	Particle Swarm Optimization
SDPSO	Strategy Dynamics Particle Swarm Optimization
SI	Swarm Intelligence
VPPSO	Velocity Pausing Particle Swarm Optimization
